# Metabolomic profiles of dietary exposure associated with frailty in older adults: A systematic review

**DOI:** 10.1016/j.tjfa.2026.100163

**Published:** 2026-06-06

**Authors:** Samar El Sherbiny, Evgeniia Puzankova, Miriam Martínez-Huélamo, Natàlia Garcia-Giralt, Jose Antonio Carnicero Carreño, Leocadio Rodriguez Mañas, Jose Antonio Serra Rexach, Francisco José García García, Xavier Nogués, Pedro Abizanda Soler, Cristina Andrés-Lacueva, Montserrat Rabassa

**Affiliations:** a Biomarkers and Nutrimetabolomics Laboratory, Department of Nutrition, Food Sciences and Gastronomy, Faculty of Pharmacy and Food Sciences, University of Barcelona, 08028 Barcelona, Spain; b Department of Public Health and Pediatrics, University of Turin, 10126 Turin, Italy; c Biomarkers and Nutrimetabolomics Laboratory, Department of Nutrition, Food Sciences and Gastronomy, Nutrition and Food Safety Research Institute (INSA-UB), Faculty of Pharmacy and Food Sciences, University of Barcelona (UB), 08028 Barcelona, Spain; d Centro de Investigación Biomédica en Red de Fragilidad y Envejecimiento Saludable (CIBERFES), Instituto de Salud Carlos III, 28029 Madrid, Spain; e Hospital del Mar Research Institute, Barcelona, Spain; f Fundación para la Investigación Biomédica del Hospital Universitario de Getafe, Instituto de Investigación Sanitaria Hospital Universitario de Getage (IISGetafe), Spain; g Hospital General Universitario Gregorio Marañón, Madrid, Spain; h Complejo Hospitalario de Toledo - SESCAM, Spain; i Complejo Hospitalario Universitario de Albacete, Spain

**Keywords:** Diet, Food, Nutrition, Metabolomics, Metabolites, Biomarkers, Frailty

## Abstract

Frailty is defined by the World Health Organization as a state of increased vulnerability in older adults, characterized by a decline in physiological functions across multiple systems and heightened sensitivity to external stressors. The global prevalence of frailty is expected to rise due to population ageing, highlighting the need for effective preventive strategies. However, while diet can play a crucial role in this context, their relationship is complex and potentially bidirectional: unhealthy dietary patterns may contribute to the onset of frailty, whereas early stages of frailty may influence food choices through limitations in meal preparation, and appetite. Hence, identifying biomarkers associated with frailty phenotypes may help clarify these mechanisms and improve prevention strategies. This systematic review aims to summarize current evidence on metabolomics-derived biomarkers potentially involved in the relationship between dietary patterns and frailty. A comprehensive literature search was conducted across three electronic databases (PubMed, Web of Science, Scopus) in accordance with the PRISMA guidelines, yielding 1661 studies, of which five met the inclusion criteria. Three dietary exposures were identified and analyzed: fruit and vegetable, plant/animal protein intake, and adherence to the Mediterranean diet. The findings suggest that specific metabolites, such as hippuric acid, different amino acids, and lipid derived compounds may represent potential candidates for establishing a metabolic signature of frailty. Nevertheless, current evidence remains limited and unclear about the direction of associations. Further research is needed, particularly in healthy populations, to validate the effectiveness of these biomarkers, and tailor guidelines specific for older adults.

## Highlights

• Mediating metabolites can originate not only from dietary exposure but also from the microbiota.
• Presence of metabolites on multiple metabolic pathways hints a complex role of dietary exposure.
• Gender may influence predictive metabolites of frailty.
• Metabolomics for biomarker discovery is crucial in tailoring dietary guidelines for older adults.

## 1 Introduction

The global population aged 60 years or older is estimated to double by 2050 [1] and reach its peak in the late 2070 [2]. Recent studies indicate that the global frailty among older adults varies widely, ranging from 4.9 % to 65.2 %, with 5.2 % to 19.1 % of those affected being aged 65 years or older [3,4]. Frailty is defined by the World Health Organization (WHO) as a state of increased vulnerability, characterized by a decline in physiological function across multiple systems and heightened sensitivity to stressors, and is associated with adverse health outcomes such as falls, disability, hospitalization, and mortality [[5], [6], [7]]. Although no universal definition exists, frailty is widely recognized as a dynamic and potentially reversible condition. The most used assessment models include the frailty phenotype [8] and the frailty index (FI) [9,10]. Reduced mobility, typical of frailty, often leads to a high level of dependence of the older adults on external care, which reduces the quality of life of people that ended up with poor mental health, reduced social engagement and phycological distress [1].

Several modifiable factors contribute to the onset or progression of frailty, including clinical, lifestyle, demographic, and socioeconomic elements. Among these, frailty often overlaps with malnutrition [11], and for this reason, dietary interventions are particularly significant, as poor nutritional status adversely affects all five criteria of the frailty phenotype [6,12]. Numerous observational studies, both with a cohort and cross-sectional design, have demonstrated the efficacy of specific dietary patterns in mitigating frailty, including anti-inflammatory diets [13,14] and the Mediterranean Diet (MedDiet), which has been associated with a reduced incidence of frailty [15]. Key components of the MedDiet such as fruits, vegetables, legumes, nuts, seeds, and olive oil are rich in phytonutrients like polyphenols, known for their potent antioxidant and anti-inflammatory properties, making this dietary pattern particularly effective in reducing the risk of pre-frailty and frailty in older adults [16,15,12]. Several meta-analyses further support these findings [17,18].

Despite the extensive body of literature exploring the relationship between frailty and nutrition, frailty remains a multifactorial condition, and the identification of molecular biomarkers to diagnose and monitor frailty or pre-frailty is still an unmet need. Metabolomics is one of the omics sciences that represents a promising approach to address this gap [[12], [19], [20]], also due to its ability to simultaneously detect hundreds of metabolites in relation to the exposome [21]. More in detail, Nutrimetabolomics could be a powerful tool that can identify biological mechanisms that are influenced by nutrients [22] and can uncover the relationship between dietary patterns and frailty. The identification of proper nutritional biomarkers that can be used to detect frailty or pre-frailty phenotype early is fundamental as a public health prevention strategy not only for the onset of frailty but also to reduce the burden that this condition has on healthcare systems [6].

However, to date, no comprehensive synthesis has examined the mediating role of diet-derived metabolites in the development of frailty. Identifying groups of these metabolites, rather than single compounds, may help define metabolic signatures associated with healthy dietary patterns, thereby providing insight into the mechanisms linking dietary exposures to frailty. These metabolic signatures may help identify individuals at higher risk of pre-frailty and transition to frailty. Therefore, the objective of the present review is to systematically summarize current evidence on metabolites identified through metabolomics that mediate the relationship between healthy dietary patterns and frailty risk in older adults.

## 2 Material and methods

The present work was conducted in accordance with the Preferred Reporting Items for Systematic Reviews and Meta-Analyses (PRISMA) 2020 statement reporting checklist for systematic reviews [23] (Table S1) and based on the registered PROSPERO protocol (registration number CRD42024550465 - July 2024).

### 2.1 Data sources and search strategies

Only original papers were considered for this review. A preliminary search was conducted in the main databases to identify potential gold standard articles and relevant keywords (e.g., “diet, food and nutrition” and “Metabolomics” and “frailty”). The search strategy was then specifically tailored to each employed database, PubMed, Scopus and Web of Science, and the string launched on October 9, 2024, and updated on March 02, 2026. The complete search strategy is available in Appendix A.

### 2.2 Eligibility criteria

Inclusion and exclusion criteria were defined in the PROSPERO protocol and applied during the screening of results retrieved from the aforementioned databases. Inclusion criteria: 1) Full-text in English or Spanish, 2) randomized controlled trials and observational studies that involved humans and evaluated the consumption of, or adherence to, clearly defined dietary patterns, including, at a minimum, a description of the foods and beverages in the pattern, and of the dietary assessment method, 3) studies including older adults people living in the community(age ≥ 60 years), healthy and/or at risk of chronic disease and/or obese, or studies in which a control group of healthy people with the aforementioned characteristics was present, 4) presence of metabolomic biomarkers of dietary patterns, and 5) investigation of a possible correlation between frailty status, dietary patterns, and metabolomic signatures. Studies should clearly define frailty, and the assessment method used. Reviews, expert opinions, editorials, conference abstracts, unpublished data, and pre-prints are excluded. Additionally, the absence of a detailed description of the dietary intake and the absence of metabolomic methodology for biomarker assessment determines the exclusion of the articles. Studies that did not consider frailty or prefrailty as the primary outcome of interest were excluded. Three independent authors conducted the screening process of the selected works from the title to the full text. Mendeley software (version 2.122.1) was used to organize, and screen identified articles.

### 2.3 Data extraction

Data were extracted from one author and recorded in an Excel spreadsheet. The extracted information included the study design (type, duration, country, cohort name, objectives, and main findings, including statistical methods and approaches used to evaluate the associations of interest), population characteristics (sample size, age range, sex, and baseline status (e.g., health information)), dietary assessment (assessment tool and dietary pattern considered), metabolomic profile (biospecimen type, biomarker assessment method, number and type of biomarkers reported), and frailty assessment details. If the authors specified that some part of the study was already explained in previous work, the information was taken from previous publications. After this process, a second author checked all the data, and any disagreement was discussed.

### 2.4 Quality assessment

The evaluation of the risk of bias (RoB) was performed by two independent authors using an appropriate checklist. The tool employed was the Nutrition QUality Evaluation Strengthening Tools (NUQUEST) [24], a specific tool for evaluating RoB in nutritional studies. All discrepancies were discussed and clarified with a third author. The evaluation of RoB was expressed as a percentage and the final score was recoded based on tertiles (1st tertile 0–33 % = poor quality; 2nd tertile 34–66 % = medium quality; 3rd tertile 67–100 % = high quality) [25].

### 2.5 Data synthesis and analysis

A narrative synthesis of the findings from the included studies was performed. Due to the heterogeneity in the included studies, a meta-analysis was not feasible.

## 3 Results

### 3.1 Search and selection of studies

**Fig. 1:**
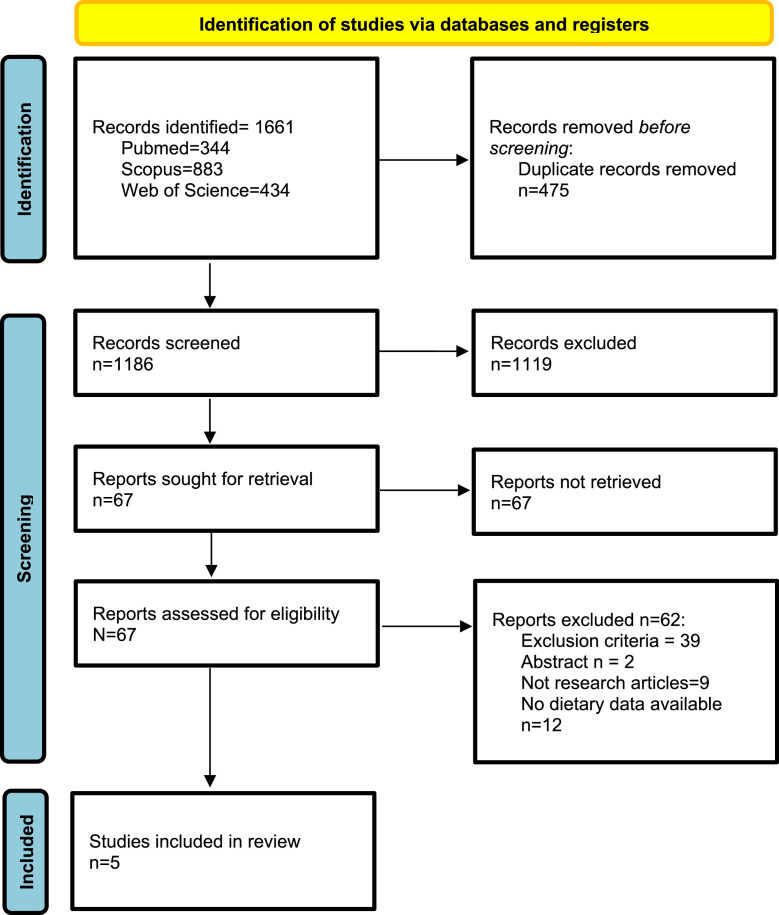
PRISMA flow diagram of studies selection (modified from Page et al [23]).

The search and selection process of the studies is summarized in detail in a flow diagram (Fig. 1). A total of 1661 references were identified through the initial search strategy, which became 1186 after the removal of duplicates. After screening titles and abstracts, 1119 references were excluded for not meeting the predefined inclusion criteria. Common reasons included focusing solely on one specific component of frailty, not describing dietary patterns in detail, not including metabolomic assessments, not investigating the connection between the metabolomic profile and the relationship between frailty and dietary patterns, or an age range that included population younger than 60 years. (Table S2). Consequently, 5 studies [[26], [27], [28], [29], [30]], were deemed eligible for this systematic review.

### 3.2 Studies characteristics and methods

The main characteristics of the selected studies are presented and summarized in Table 1. In the present systematic review, three cohort studies were included [[28], [29], [30]], one observational study [27] and a longitudinal population-based cross-sectional study [26]. All the included studies were conducted in high income countries, with two studies conducted in the USA [29,30], one in Poland [27], one in Italy [26] and one multicentric study conducted in Italy and Poland [28]. All five studies included data on both men and women and focused on adult populations, specifically older adults, with a sample size ranged from 29 to 806. Mean age range goes from 69.3 ± 5.3 years to 77± 7.5 years. Two out of five studies performed a follow-up: the BLSA cohort includes different follow-up times, but in the studies of Tanaka et al [30] and Tanaka et al [29], only a single time point was considered. Laura Brunelli et al. and Estelle Pujos-Guillot et al. had, respectively, two follow-up times after 4 and 8 years, and time point after 1 year. Finally, the study of Alina Jaroch et al. does not have a follow-up.

**Table 1:** Summary of the main characteristics of the included studies.

Reference	Study design and duration	Country	Sample size ( % Female)	biospecimen	Dietary exposure/assessment tool	Frailty assessment tool	RoB
[27]	observational prospective study 2015–2017	Poland	45 (29 robust, 82 %)	blood serum	Total protein intake 24 h recall	robust, pre-frail or frail using the criteria developed by Fried et al.	High
[28]	NU-AGE cohort study 2011–2016	Italy and Poland	Baseline: 212 (127 robust, 53 %) Sub-cohort: 120 (60 robust, % NA)	serum	MedDiet vs. control group NU-AGE diet adherence scoring system	robust, pre-frail or frail using the criteria developed by Fried et al.	High
[29]	BLSA cohort study 1958-ongoing	USA	735 ( % reported for each quartile)	Overnight fasting plasma	Animal and plant protein consumption FFQ, MDS for diet quality	44-item frailty index by Searle et al.	Medium
[30]	BLSA cohort study 1958-ongoing	USA	806 (51,5 %)	Overnight fasting plasma	MDS, MIND, AHEI FFQ	44-item frailty index by Searle et al.	Medium
[26]	InveCe.Ab longitudinal population-based cross-sectional study 2010-ongoing	Italy	First metabolomic analysis 2014: 65 robust validation set: 124 fit (48,4 %) longitudinal 2014–2018: 2014: 110 2018: 60	plasma	fresh fruit and vegetable intake FFQ	Frailty index - based on 32 variables	High

The main healthy dietary pattern examined was the MedDiet [[28], [29], [30]] by using different indexes like the Mediterranean style diet score (MDS) [29,30], Mediterranean-DASH Diet Intervention for Neurodegenerative Delay (MIND), and Alternate Healthy Eating Index (AHEI) [30]. Other healthy dietary exposures were plant- and animal-based protein intake [29], fruit and vegetable intake [26] and the total protein intake [27], each of them explored in a single study. Dietary assessment was performed using Food Frequency Questionnaires in three studies [26,29,30], Alina Jaroch et al., used a 24 h recall method, and finally the study from Estelle Pujos-Guillot et al. employed an adherence scoring system based on the NU-AGE Food Based Dietary guidelines.

Frailty status of the participants was assessed using the Frailty Index (FI) as defined by Searle et al. in two studies [29,30], and the Frailty Phenotype defined by Fried et al [8] in two other studies [[27], [28]]. The study of Laura Brunelli et al. used a list of 32 health variables or deficits to determine the Frailty degree of the patients.

Two studies utilized fasting plasma samples [29,30] while the study by Laura Brunelli et al [26], did not specify the fasting status of plasma collection. The remaining two studies used serum samples [[27], [28]], collected after overnight fasting in the case of Estelle Pujos-Guillot et al [28], while in the study of Jaroch and colleagues it was collected during a convenient time of the day, non-fasting. Methodologies for the detection of those metabolites involved an untargeted metabolomics approach, UPLC-MS in the study by Estelle Pujos-Guillot et al.. In two studies, Flow Injection Analysis–Tandem Mass Spectrometry (FIA-MS/MS) was used to analyze targeted lipids and hexoses in fasting plasma [29,30]. Furthermore, Liquid Chromatography–Tandem Mass Spectrometry (LC-MS/MS) was applied in four studies for targeted metabolite profiling [[26], [27], [29], [30]].

The included studies used diverse statistical approaches to evaluate the associations of interest (Table S3). More in detail Tanaka et al [30] represented the results as elastic net regression coefficients and correlation coefficients, Tanaka et al [29] used a multiple linear regression model, and a mediation analysis, Brunelli et al [26] used a linear regression model, and a mediation analysis following the Baron and Kenny method. Jaroch et al [27] used parametric and non-parametric tests in order to assess the differences between the groups and finally Pujos-Guillot et al [28] used a multivariate logistic regression model.

### 3.3 Studies quality assessment

Overall, the score was high for all the studies (Table 1). With the NUQUEST scale three studies were classified as “medium quality” [[28], [29], [30]] while two as “high quality” [[26], [27]]. The main weaknesses identified were an overall lack of sample size justification or power description, only one study [26] provided information about the participation rate. In most of the studies the follow up was not taken into consideration in the analysis. Regarding the nutrition-specific questions, none of the studies performed multiple measurements of the diet, hence a diet change monitoring was not operated, to check if the differences among the groups were maintained.

### 3.4 Metabolomic profiles associated with dietary exposure

Table 2 shows a list of the biomarkers related to diet detected with metabolomic methodologies, that exhibit a relationship with frailty onset. In the BLSA cohort study by Tanaka et al [30], which investigated metabolomic profiles associated with different dietary patterns and their relationship to the Frailty Index, a total of 466 metabolites were assessed. Of these, 176 metabolites were significantly associated with the three dietary scores MDS, MIND, and AHEI analyzed in this study. These metabolites included tri- and di- glycerides, cholesteryl ester, ceramides, lysophosphatidylcholines, sphingomyelins, amino acid-related compounds, bile acids, fatty acids, indoles and derivatives, acylcarnitines, and carboxylic acids. More in detail, the most positively correlated with diet scores were triglycerides (22:6_34:3), and a cluster of highly correlated metabolites was identified having a negative association with the analyzed scores (phosphatidylcholine diacyl C40:4 was the most significant); however, no coefficients or p-values (quantitative measures) were reported for these specific metabolites. The discriminant metabolites are reported in Table 2.

**Table 2:** Summary of the main characteristics of the included studies, including the discriminant metabolites, main methodologies and main findings.

Reference	Dietary exposure	Matrix	Biomarker N	Biomarkers	Analytical method	Increasing metabolites	Decreasing metabolites	Covariates	Main findings - frailty association
[27]	Proteins	serum	31 candidates from untargeted metabolomic analysis 5 metabolites significantly differed between the groups	Arachidonic acid Oleoylethanolamide Taurine Methionine Leucine	SPME assay+LC-MS	Arachidonic acid Taurine Methionine Leucine	Oleoylethanolamide	BMI body weight protein consumption MMI	Metabolomic assessment showed that nutritional intervention caused a significant change in three compounds—arachidonic acid (ARA), oleoylethanolamide (OEA), and valine. The increase in 5 metabolites associated with a diet rich in proteins lead to the restoration of a phenotype similar to the robust one
[28]	MedDiet	serum	4 for each gender identified as predictive for prefrail status 1 fro each gender mediate the relationship between diet and frailty	Associated with frailty: men: glutamine gly-phe dimethyloxazole mannose women: threonine fructose mannose N-(2-hydroxypropyl)-valine	Untargeted metabolomics UPLC-MS	NA	NA	country	4 metabolites for each gender were selected as predictive markers for prefrail status with good performance 2 metabolites mediated the relationship between diet and frailty No significant results with the dietary patterns
[29]	Plant proteins	plasma	466 passed the quality control 25 associated with plant protein consumption 15 metabolites mediate the association between plant protein consumption and frailty index	cholesterol esters (4), sphingomyelins (4), phosphatidyl-cholines (6), and 3IPA	(LC–MS/MS FIA-MS/MS for lipids and hexose MS and MetIDQ sw for metabolites quantification	3IPA	cholesterol esters, sphingomyelins, phosphatidyl-cholines	age, sex, BMI, self-reported race, smoking status, calendar year of BLSA visit, total energy intake, % energy from total fat, alcohol intake (g/day)	Consumption of plant protein (as % energy) was negatively associated with FI, while animal protein and total protein intake were not associated with FI. . 15 metabolites mediated the association between plant protein intake and FI. The protective association between plant protein consumption and FI is mediated by lower abundance of lipid metabolites and higher abundance of tryptophan-related metabolites.
[30]	MedDiet	plasma	466 passed the quality control 236 metabolites associated with the MDS 218 metabolites associated with MIND 278 metabolites associated with AHEI 176 associated with all three scores 4 mediate the association between diet and frailty index	TG(22:6_34_3) most significant positively associated with all 3 dietary scores PC diacyl C40:4 most significant negatively associated with all 3 dietary scores Metabolites selected for mediation analysis: tryptophan betaine (TrpBetaine), CE (17:1), PCaaC40:5, and PCaeC42:3 - associated with all 3 dietary scores	LC–MS/MS FIA-MS/MS for lipids and hexose MS and MetIDQ sw for metabolites quantification	NA	NA	age, sex, BMI serum creatinine eGFR	-TrpBetaine, CE(17:1), PCaa C40:5, and PCae C42:3 that were common to all three diet pattern summary scores.-Metabolomic signatures of diet explained 28 %, 37 %, and 38 % of the variance of the MDS, MIND, and AHEI-Signatures of MIND and AHEI mediated 55 % and 61 % of the association between each dietary pattern with FI
[26]	fruit and vegetables	plasma	43 metabolites retrieved with targeted metabolomics (mainly lipid species) 22 metabolites retrieved with untargeted metabolomics who changed significantly between frail and fit population 1 metabolite differed significantly between the groups and mediated the relationship between frailty index and diet	Hippuric acid Bilirubin Tryptophan Arginine 1-Stearoylglycerophosphoglycerol phosphatidylserine(1) sphingomyelins (3), phosphatidyl-cholines (4), Di/Triglycerides (3), Lyso-phosphatidyl-cholines (3), Phosphatidylglycerols (3)	LC-MS	/	Hippuric acid	hippuric acid level age, education, gender	-low plasmatic hippuric acid as a plausible hallmark of frailty status associated with a decline in Fruit–Veg intake -high hippuric acid may be associated with a lower risk of frailty - 22 metabolites whose change in abundance differed significantly between frail and fit populations

The study by Tanaka et al [29], explored the relationship between protein intake and frailty. They identified 466 plasma metabolites, and out of these, 25 metabolites were found to be associated with quartiles of plant protein consumption after adjusting for covariates. Higher plant protein consumption was associated with lower levels of several lipid metabolites, while higher plant protein intake was associated with increased levels of tryptophan betaine (TrpBetaine: β=0.24, *p* = 1,14E-10) and indole-3-propionic acid (3IPA: β=0.13, *p* = 0.0004). After further adjustment for MDS, metabolites such as TrpBetaine (β=0.21, *p* = 1,33E-07), some phosphatidylcholine (e.g. PC ae C42:5 β=−0.15, *p* = 0,0003) and cholesteryl ester (β=−0.15, *p* = 0,0003) continued to show significant associations with plant protein intake.

Finally, in the work of Jaroch et al [27], a nutritional intervention based on increased high quality protein consumption was tested to verify its potential impact on pre-frail patients, in order to drive them back towards a robust phenotype. They discovered a difference in pre-frail patients that finishes the nutritional intervention. The untargeted metabolomic analysis revealed 31 candidates among which arachidonic acid, oleoylethanolamide, taurine, methionine, and leucine changed significantly after the intervention (*p* = 0.037, *p* = 0.002, *p* = 0.068, *p* = 0.075, *p* = 0.066 respectively), exceeding the values reported in robust population.

### 3.5 Metabolomic profiles associated with dietary exposure and frailty

To deepen the relationship between dietary patterns and frailty, here is reported the metabolomic signature of their association (Table 2). In the study of Tanaka et al [30], four metabolites were associated with the three dietary scores MIND, MDS, and AHEI (*R* = 0.49, *R* = 0.42, *R* = 0.56, respectively), 19 metabolites associated with two dietary patterns, and 60 metabolites to a single dietary pattern. They conducted a mediation analysis to determine whether the metabolomic diet score mediated the relationship between two of the analyzed dietary patterns (MIND and AHEI) and the frailty index (FI). The analysis revealed that the metabolomic diet score mediated 55 % of the association between MIND and frailty and 61 % of the association between AHEI and frailty.

Brunelli et al [26], focused on the metabolites associated with frailty rather than starting from the diet. To identify a strong difference between robust and frail populations, an untargeted metabolomic analysis was performed and 22 metabolites were identified that differed significantly between the groups. The metabolite that differed mainly was hippuric acid, which was 2 times lower in frail patients than in a robust population (Fold Change −1.8). Moreover, hippuric acid shows a significant inverse relationship with the FI (β =−0.156; *p* = 0.007). Finally, a mediation analysis was performed to determine the possible role of fruit and vegetable consumption (the main dietary pattern investigated in this study). The results supported the mediating role of fruit and vegetables in hippuric acids levels (β=−0.113, *p* = 0.06), supported by the analysis on the follow-up after 4 years, in which the risk of frailty was reduced. Tanaka et al [29], conducted a mediation analysis regarding the relationship between plant protein and frailty index. Their results highlighted that consumption of plant protein (as % energy) was negatively associated with FI, while the intake of animal protein and total protein was not associated with FI. Out of the 25 metabolites associated with plant protein consumption, 15 partially mediated the association with FI with a percentage of mediation between 8.5 % and 16.6 %. The mediating metabolites included: cholesterol esters, sphingomyelins, phosphatidylcholines, and 3IPA. Finally, Pujos-Guillot et al [28], analyzed the effect of MedDiet on frailty to identify the best model able to discriminate between different sub-types of pre-frailty at gender level. Untargeted metabolomics approach resulted in the identification of a set of metabolites for each gender that could be used as predictive markers of prefrail status, since they significantly differ from non-frail to pre-frail (Table 2). Regarding the possible mediating role of diet, of the 8 metabolites identified, one for each gender has been identified as modulated by diet and also predictive of pre-frail status (dimethyloxazole (*p* = 0.05) in men and fructose (*p* = 0.04) for women after the ANOVA diet vs. control). Apart from these considerations, no significant results were found regarding the relationship with the dietary pattern examined (MedDiet and NU-AGE). The authors performed correlations between serum nutrient levels and these metabolites, but no significant relationships were observed, neither with the NU-AGE diet compliance score.

## 4 Discussion

Our systematic review summarizes the current knowledge on the relationship between healthy dietary patterns and frailty, with a particular focus on dietary-derived metabolites that may mediate this association. We explored different dietary components, including protein intake, fruit and vegetable intake, and MedDiet, in relation to frailty and pre-frailty phenotypes compared to robust population. After a comprehensive literature search, only five studies met the inclusion criteria, highlighting the limited but growing body of evidence in this topic. The included studies demonstrated heterogeneity in both methodology and populations, underlining the complexity of identifying a consistent metabolic signature predictive of frailty. One of the main limitations encountered was the scarcity of longitudinal studies involving robust populations over 60 y.o., which limits the ability to identify early effect biomarkers. Hence, this systematic review should be a starting point to guide future research in this topic. In recent years, the use of metabolomics to investigate frailty has gained attention. Given the well-established burden of frailty on healthcare systems [6], elucidating the metabolomic profile associated with frailty and its dietary determinants is essential for improving prevention strategies in public health [31].

The results summarised from this systematic review highlight the potential of a healthy diet to delay or prevent the onset of frailty. Recent studies support the interaction between dietary patterns, such as the Mediterranean diet, and gut microbiota in shaping early mechanisms of frailty [32]. However, the lack of studies among robust older adults limits the ability to identify reliable early biomarkers. Moreover, rather than focusing on single foods or nutrients, future research should emphasize overall dietary patterns, as the synergy among different foods and nutrients is known to have a strong effect on health [33].

Two of the included studies investigated the effect of protein intake and protein quality on frailty onset and progression [27,29]. Both studies identified mainly amino acids or compounds derived from amino acids metabolites potentially involved in improving the frailty phenotype. Jaroch et al [27] identified some amino acid profiles in pre-frail participants that suggested restoration beyond robust levels. Tanaka et al [29] found that plant protein-derived metabolites were negatively associated with the FI, whereas animal or total protein intake showed no such association. The authors explained this by the overall reduction in lipid intake observed in participants with higher plant protein consumption. This reduction likely reflects a lower intake of animal-derived fats, typically richer in saturated fatty acids with potential pro-inflammatory effects, reinforcing the importance of dietary context and quality over isolated nutrients. The role of protein in frailty and sarcopenia is well established, particularly in the context of anabolic resistance, which makes it more difficult to maintain muscle mass and the balance between muscle protein synthesis and breakdown that occurs with aging, thereby increasing protein requirements [34]. Inadequate protein intake may accelerate muscle loss, leading to physical decline, sarcopenia and frailty [35]. Diet can be the solution to prevent this kind of decline [36,37]. While animal proteins can stimulate muscle synthesis in older populations [38,34], plant proteins have been linked to healthy aging, partly due to their role in displacing less favourable dietary components, such as processed meats and animal-derived foods high in saturated fats, often associated with adverse health outcomes and many causes of mortality [39,40]. This shift may contribute to an overall improvement in dietary quality, aligning with the results from Tanaka et al [29]. This makes it even more important to identify biomarkers that allow us to evaluate the diet of older adults to improve the frail phenotype, and to tailor guidelines specific to older populations, whose nutritional needs differ from those of younger adults [36]. Notably, frailty can result from multiple pathways, including sarcopenia, hospitalization, cognitive decline, cardiovascular diseases, renal or immune dysfunction, among others, which may ultimately converge into similar clinical phenotypes. This diversity of underlying mechanisms highlights the potential role of personalized nutrition strategies and metabolomics to improve the precision and effectiveness of dietary interventions in older adults [41]. Regarding the intake of fruit and vegetables, only one study deepens this food group in our review [26]. Brunelli et al [26] identified that hippuric acid was the metabolite that mediates the relationship between a higher level of consumption of fruits and vegetables and frailty. Hippuric acid is a well-recognized gut microbial-derived metabolite and has been proposed as a biomarker of healthy dietary patterns, particularly those rich in polyphenols. Urpi-Sarda et al [42] demonstrated that higher adherence to a polyphenol-rich diet was associated with a reduced risk of frailty and pre-frailty in community-dwelling older adults. Different studies proposed hippuric acid, one of the final microbial metabolites of dietary polyphenol biotransformation, as a dietary predictive biomarker for frailty onset [21,43] related to polyphenol consumption. Thus, while hippuric acid emerges as a promising candidate representative of the polyphenol category, further research is needed to identify additional metabolites with similar mediating roles, particularly those linked to fruit and vegetable intake. Moreover, given the central role of the gut microbiota in polyphenol metabolism and its interaction with diet, future strategies could aim to modulate microbiota composition through dietary interventions, such as adherence to the MedDiet, to delay or mitigate frailty progression [44,32]. The literature is full of evidence that proved the benefits of this dietary pattern, but the identification of specific biomarkers is difficult. In the studies of Tanaka et al [30], and Pujos-Guillot et al [28] the research related to mediterranean dietary pattern led to the identification of different metabolites that have a mediating role between diet and frailty. More in detail, Tanaka et al [30] found that most of the metabolites mediating the effects of the Mediterranean diet are lipids or lipid-derived compounds. Pujos-Guillot instead focus on the potential influence that covariates like gender can have on frailty onset and progression. Different studies proved the higher incidence and prevalence of frailty in women [[45], [46], [47]]. This suggests a need to develop gender-specific metabolic signatures of frailty. Overall, a wide range of lipids species, amino acids and derivatives, bile acids, and sugars mediated the relationship between diet and frailty in the studies here summarised. Notably, several of these mediating metabolites originate from gut microbial metabolism (e.g., hippuric acid, 3IPA), emphasizing the critical and often underestimated interplay between diet, gut microbiota, and frailty, as reported in the NU-AGE study [48]. The differences in gut microbiome signature between frail and non-frail populations has been observed also in other studies [49]. A review by Ticinesi et al [50] also emphasized the possible role of gut microbiota on the onset of malnutrition, frailty and sarcopenia. While evidence supporting the benefits of healthy dietary patterns on frailty continues to grow, there is still work to do. In a study by Dominguez et al [15], a strong association between MedDiet and a lower incidence of frailty has been identified, while Zhao Yao et al [14] identified several metabolite-based signatures of frailty across different dietary patterns. The identification of multiple metabolite classes derived from multiple metabolic pathways suggests that the effect of diet on frailty is highly complex. In fact, the relationship between diet and frailty is not unilateral: while healthier dietary patterns are associated with reduced risk and progression of frailty [51], the presence of the frail condition often imply poorer dietary intake, reduced appetite, and functional limitations that negatively impact food choices and nutritional status, creating a complex feedback loop between nutritional behavior and frailty development [52], [53]. In this context, together with dietary patterns, other influencing factors (like gender, physical activity, and social environment) must be considered. All the aforementioned exposures can be resumed under the concept of the exposome—the totality of environmental exposures across the lifespan— which plays a crucial role in shaping health trajectories during ageing [54]. Integrating exposome data with metabolomics may help disentangle the interplay between diet and frailty. In this context, the hallmarks of aging provide a valuable biological framework to investigate how nutrition modulates molecular aging processes and contributes to the onset or prevention of frailty [55].

Our review presents several strengths. The protocol has been registered on the PROSPERO platform, using a rigorous and explicit methodology. We adopted a sensitive and comprehensive literature search strategy and an explicit eligibility criterion. Each step of the screening was performed in duplicate, from the study selection to the data extraction and the data evaluation. Our review also included experts who have extensive experience in the fields of evidence-based nutrition, metabolomics, and frailty. However, our review has several limitations. Only five studies met the inclusion criteria, and although the methodologies and population characteristics were relatively consistent (thanks to the strict inclusion and exclusion criteria), differences in study design and analytical methods may still introduce inconsistencies and affect comparability. A comparative analysis of the results was also not feasible due to heterogeneity in the dietary exposures examined across studies. Variability in the methods used to assess diet quality may have further influenced the findings. Additionally, the studies employed different metabolite measurement techniques and analyzed diverse sets of metabolites, underscoring the need for more standardized approaches in future research. Another important limitation was the strict age criterion used for study inclusion, which led to the exclusion of many studies targeting younger populations (e.g., 40–45 years). Given that much research on frailty prevention focuses on these earlier age groups to delay the onset of frailty, this criterion may have limited the comprehensiveness of our review.

## 5 Conclusion

Our systematic review suggests the potential of metabolomics to create metabolic signatures of specific dietary patterns, which may mediate the relationship between dietary patterns and frailty. These metabolomic signatures could serve as valuable tools for metabotyping, allowing the identification of individuals – or subgroups – at higher risk of developing frailty. This approach not only facilitates personalized nutritional advice and dietary interventions aimed at preventing frailty but also allows the implementation of such strategies at the group level, as we highlighted for gender differences, which is crucial for public health, and to strengthen prevention strategies. In conclusion, the metabolites identified in this systematic review, particularly hippuric acid, represent a promising starting point as candidate biomarkers for defining a metabolic signature of frailty. However, further research is necessary to validate these findings and assess their clinical relevance within a broader metabolic context.

## Funding sources

This work was supported by the NUTRIFRAIL project (PID2022-141067OA-I00) funded by MICIU/AEI/10.13039/501100011033 and FEDER, EU. Additional support was provided by the 10.13039/501100004587Instituto de Salud Carlos III through the CIBER of Frailty and Healthy Aging (CIBERFES, CB16/10/00269), and by the INSA-UB Maria de Maeztu Unit of Excellence grant (CEX2021-001234-M), funded by MCIU/AEI/FEDER, EU.

## Ethic approval

No approval was needed because the data was obtained from secondary sources.

## Data statement

This systematic review did not generate or analyse new primary data. All data supporting the findings of this study are derived from previously published articles, which are cited in the reference list and reported within the article.

## Declaration of the use of generative AI and AI-assisted technologies in scientific writing and in figures, images and artwork

Authors declare that no generative artificial intelligence (AI) or AI-assisted technologies were used in the writing of this manuscript or in the preparation of figures and images.

## CRediT authorship contribution statement

**Samar El Sherbiny:** Writing – original draft, Methodology, Investigation, Formal analysis, Data curation. **Evgeniia Puzankova:** Formal analysis. **Miriam Martínez-Huélamo:** Writing – review & editing. **Natàlia Garcia-Giralt:** Writing – review & editing. **Jose Antonio Carnicero Carreño:** Writing – review & editing. **Leocadio Rodriguez Mañas:** Writing – review & editing. **Jose Antonio Serra Rexach:** Writing – review & editing. **Francisco José García García:** Writing – review & editing. **Xavier Nogués:** Writing – review & editing. **Pedro Abizanda Soler:** Writing – review & editing. **Cristina Andrés-Lacueva:** Writing – review & editing. **Montserrat Rabassa:** Writing – review & editing, Writing – original draft, Visualization, Validation, Supervision, Software, Resources, Project administration, Methodology, Investigation, Funding acquisition, Formal analysis, Data curation, Conceptualization.

## Declaration of competing interest

The authors declare the following financial interests/personal relationships which may be considered as potential competing interests:

Montserrat Rabassa reports a relationship with University of Barcelona that includes: employment. There are no additional relationships or activities to declare. If there are other authors, they declare that they have no known competing financial interests or personal relationships that could have appeared to influence the work reported in this paper.

## Appendix Supplementary materials

Image, application 1
