# Supplementary material for: Metabolomic profiles of dietary exposure associated with frailty in older adults: A systematic review
Source: J Frailty Aging. 2026 Jun 6;15(4):100163. doi: 10.1016/j.tjfa.2026.100163 (PMC13264361; doi:10.1016/j.tjfa.2026.100163)
Supplement: Supplementary file 1 [file mmc1.docx]

**Supplementary material**

**Appendix A**

# **PubMed**

("Frail Older adults"[Mesh] OR "Frailty"[Mesh] OR frail*[tiab] OR prefrail*[tiab] OR ("Aged"[Mesh] OR aged[tiab] OR elder*[tiab] OR older[tiab] OR geriatr*[tiab] OR gerontol*[tiab] AND fragil*[tiab])) AND ("Metabolomics"[Mesh] OR "Metabolome"[Mesh] OR metabolom*[tiab] OR metabonomic*[tiab] OR metabolit*[tiab] OR "metabolic profile*"[tiab] OR secretom*[tiab] OR lipidom*[tiab] OR nutrimetabolom*[tiab] OR "metabolic marker*"[tiab] OR "metabolic biomarker*"[tiab] OR "metabolic bio-marker*"[tiab] OR "Ergothioneine"[Mesh] OR ergothionein*[tiab] OR thionein*[tiab] OR thiasin*[tiab] OR thiazin*[tiab] OR "alpha-Tocopherol"[Mesh] OR alpha-tocopherol*[tiab] OR a-tocopherol*[tiab] OR alphatocopherol*[tiab] OR tocopherol-acetate[tiab] OR tocopheryl-acetate[tiab] OR "hippuric acid"[Supplementary Concept] OR "hippuric acid"[tiab] OR hippurate[tiab] OR "benzamidoacetic acid"[tiab] OR benzoylglycine[tiab] OR "2,3-bis(3'-hydroxybenzyl)butyrolactone"[Supplementary Concept] OR enterolactone[tiab] OR "Lycopene"[Mesh] OR lycopen*[tiab] OR "Lutein"[Mesh] OR lutein*[tiab] OR xanthophyl*[tiab] OR "Beta-Cryptoxanthin"[Mesh] OR beta-cryptoxanthin*[tiab] OR "beta Carotene"[Mesh] OR beta-caroten*[tiab] OR betacaroten*[tiab] OR b-caroten*[tiab] OR "alpha-carotene"[Supplementary Concept] OR alpha-caroten*[tiab] OR alphacaroten*[tiab] OR carotene-alpha[tiab] OR "stachydrine"[Supplementary Concept] OR "proline betain*"[tiab] OR stachydrin*[tiab] OR dimethylprolin*[tiab] OR "Phytoestrogens"[Mesh] OR phytoestrogen*[tiab] OR phyto-estrogen*[tiab] OR plant-estrogen*[tiab] OR phytooestrogen*[tiab] OR phyto-oestrogen*[tiab] OR plant-oestrogen*[tiab] OR "Isoflavones"[Mesh] OR isoflavon*[tiab] OR homoisoflavon*[tiab] OR "Lignans"[Mesh] OR lignan*[tiab] OR neolignan*[tiab] OR "Phenols"[Mesh] OR phenol*[tiab] OR polyphenol*[tiab] OR "caffeic acid"[Supplementary Concept] OR "caffeic acid"[tiab] OR caffeate[tiab] OR "Flavonoids"[Mesh] OR flavonoid*[tiab] OR bioflavonoid*[tiab] OR "Beta-Cryptoxanthin"[Mesh] OR beta-cryptoxanthin*[tiab] OR b-cryptoxanthin*[tiab] OR "Genistein"[Mesh] OR genistein*[tiab] OR genestein*[tiab] OR "daidzein"[Supplementary Concept] OR daidzein*[tiab] OR daidzein*[tiab] OR "pentadecanoic acid*"[Supplementary Concept] OR "pentadecanoic acid*"[tiab] OR 9-Hexadecenoic-acid*[tiab] OR "palmitelaidic acid"[tiab] OR 9-trans-Hexadecenoic-acid*[tiab] OR "Acetylcarnitine"[Mesh] OR acetylcarnitin*[tiab] OR acetyl-carnitin*[tiab] OR acetyl-L-carnitin*[tiab] OR "Hydroxyproline"[Mesh] OR hydroxyprolin*[tiab] OR oxyprolin*[tiab] OR "3-methylhistidine"[Supplementary Concept] OR methylhistidin*[tiab] OR 3-carboxy-4-methyl-5-propyl-2-furanpropanoic[tiab] OR CMPF[tiab] OR trimethylamine-N-oxide[tiab] OR TMAO[tiab] OR "Docosahexaenoic Acids"[Mesh] OR docosahexaeno*[tiab] OR docosahexeno*[tiab] OR DHA[tiab] OR "2,3-bis(3'-hydroxybenzyl)butane-1,4-diol"[Supplementary Concept] OR enterodiol*[tiab] OR alkylresorcinol*[tiab] OR resorcinol*[tiab] OR dihydroxybenzoic*[tiab] OR DHBA[tiab] OR hydroxytyrosol[tiab] OR hydroxy-tyrosol[tiab] OR "Ethanolamine"[Mesh] OR ethanolamin*[tiab] OR colamin*[tiab] OR monoethanolamin*[tiab] OR aminoethanol*[tiab] OR amino-ethanol*[tiab] OR "Urea"[Mesh] OR urea[tiab] OR "S-Adenosylmethionine"[Mesh] OR adenosylmethionin*[tiab] OR adenosyl-methionin*[tiab] OR adenosyl-L-methionin*[tiab] OR ademetionin*[tiab] OR "dimethylglycine"[Supplementary Concept] OR dimethylglycin*[tiab] OR "trimethylamine"[Supplementary Concept] OR trimethylamine*[tiab] OR "Succinic Acid"[Mesh] OR "succinic acid*"[tiab] OR succinate[tiab] OR "butanedioic acid*"[tiab]) AND ("Diet, Food, and Nutrition"[Mesh] OR "Diet Therapy"[Mesh] OR "diet therapy"[Subheading] OR diet*[tiab] OR food*[tiab] OR beverage*[tiab] OR drink*[tiab] OR eat[tiab] OR eating[tiab] OR eaten[tiab] OR ate[tiab] OR intake[tiab] OR consum*[tiab] OR nutrit*[tiab] OR nutrient*[tiab] OR portion*[tiab])

# **Scopus**

TITLE-ABS-KEY(frail* OR prefrail* OR (aged OR elder* OR older OR geriatr* OR gerontol* AND fragil*)) AND TITLE-ABS-KEY(metabolom* OR metabonom* OR metabolit* OR "metabolic profile*" OR secretom* OR lipidom* OR nutrimetabolom* OR "metabolic marker*" OR "metabolic biomarker*" OR "metabolic bio-marker*" OR ergothionein* OR thionein* OR thiasin* OR thiazin* OR alpha-tocopherol* OR a-tocopherol* OR alphatocopherol* OR tocopherol-acetate OR tocopheryl-acetate OR "hippuric acid" OR hippurate OR "benzamidoacetic acid" OR benzoylglycine OR enterolacton* OR lycopen* OR lutein* OR xanthophyl* OR beta-cryptoxanthin* OR beta-caroten* OR betacaroten* OR b-caroten* OR alpha-caroten* OR alphacaroten* OR carotene-alpha OR "proline betain*" OR stachydrin* OR dimethylprolin* OR phytoestrogen* OR phyto-estrogen* OR plant-estrogen* OR phytooestrogen* OR phyto-oestrogen* OR plant-oestrogen* OR isoflavon* OR homoisoflavon* OR lignan* OR neolignan* OR phenol* OR polyphenol* OR "caffeic acid" OR caffeate OR flavonoid* OR bioflavonoid* OR beta-cryptoxanthin* OR b-cryptoxanthin* OR genistein* OR genestein* OR daidzein* OR daidzein* OR "pentadecanoic acid*" OR 9-Hexadecenoic-acid* OR "palmitelaidic acid" OR 9-trans-Hexadecenoic-acid* OR acetylcarnitin* OR acetyl-carnitin* OR acetyl-L-carnitin* OR hydroxyprolin* OR oxyprolin* OR methylhistidin* OR 3-carboxy-4-methyl-5-propyl-2-furanpropanoic OR CMPF OR trimethylamine-N-oxide OR TMAO OR docosahexaeno* OR docosahexeno* OR DHA OR enterodiol* OR alkylresorcinol* OR resorcinol* OR dihydroxybenzoic* OR DHBA OR hydroxytyrosol OR hydroxy-tyrosol OR ethanolamin* OR colamin* OR monoethanolamin* OR aminoethanol* OR amino-ethanol* OR urea OR adenosylmethionin* OR adenosyl-methionin* OR adenosyl-L-methionin* OR ademetionin* OR dimethylglycin* OR trimethylamine* OR "succinic acid*" OR succinate OR "butanedioic acid*") AND TITLE-ABS-KEY(diet* OR food* OR beverage* OR drink* OR eat OR eating OR eaten OR ate OR intake OR consum* OR nutrit* OR nutrient* OR portion*)

# **Web of Science**

TS=(frail* OR prefrail* OR (fragil* AND (aged OR elder* OR older OR geriatr* OR gerontol*))) AND TS=(metabolom* OR metabonom* OR metabolit* OR "metabolic profile*" OR secretom* OR lipidom* OR nutrimetabolom* OR "metabolic marker*" OR "metabolic biomarker*" OR "metabolic bio-marker*" OR ergothionein* OR thionein* OR thiasin* OR thiazin* OR alpha-tocopherol* OR a-tocopherol* OR alphatocopherol* OR tocopherol-acetate OR tocopheryl-acetate OR "hippuric acid" OR hippurate OR "benzamidoacetic acid" OR benzoylglycine OR enterolacton* OR lycopen* OR lutein* OR xanthophyl* OR beta-cryptoxanthin* OR beta-caroten* OR betacaroten* OR b-caroten* OR alpha-caroten* OR alphacaroten* OR carotene-alpha OR "proline betain*" OR stachydrin* OR dimethylprolin* OR phytoestrogen* OR phyto-estrogen* OR plant-estrogen* OR phytooestrogen* OR phyto-oestrogen* OR plant-oestrogen* OR isoflavon* OR homoisoflavon* OR lignan* OR neolignan* OR phenol* OR polyphenol* OR "caffeic acid" OR caffeate OR flavonoid* OR bioflavonoid* OR beta-cryptoxanthin* OR b-cryptoxanthin* OR genistein* OR genestein* OR daidzein* OR daidzein* OR "pentadecanoic acid*" OR 9-Hexadecenoic-acid* OR "palmitelaidic acid" OR 9-trans-Hexadecenoic-acid* OR acetylcarnitin* OR acetyl-carnitin* OR acetyl-L-carnitin* OR hydroxyprolin* OR oxyprolin* OR methylhistidin* OR 3-carboxy-4-methyl-5-propyl-2-furanpropanoic OR CMPF OR trimethylamine-N-oxide OR TMAO OR docosahexaeno* OR docosahexeno* OR DHA OR enterodiol* OR alkylresorcinol* OR resorcinol* OR dihydroxybenzoic* OR DHBA OR hydroxytyrosol OR hydroxy-tyrosol OR ethanolamin* OR colamin* OR monoethanolamin* OR aminoethanol* OR amino-ethanol* OR urea OR adenosylmethionin* OR adenosyl-methionin* OR adenosyl-L-methionin* OR ademetionin* OR dimethylglycin* OR trimethylamine* OR "succinic acid*" OR succinate OR "butanedioic acid*") AND TS=(diet* OR food* OR beverage* OR drink* OR eat OR eating OR eaten OR ate OR intake OR consum* OR nutrit* OR nutrient* OR portion*)

**Table S1. PRISMA 2020 Checklist**

| **Section and Topic** | **Item #** | **Checklist item** | **Location where item is reported** |
| --- | --- | --- | --- |
| **TITLE** | | |  |
| Title | 1 | Identify the report as a systematic review. | X (page 1) |
| **ABSTRACT** | | |  |
| Abstract | 2 | See the PRISMA 2020 for Abstracts checklist. | X (page 2) |
| **INTRODUCTION** | | |  |
| Rationale | 3 | Describe the rationale for the review in the context of existing knowledge. | X (page 3) |
| Objectives | 4 | Provide an explicit statement of the objective(s) or question(s) the review addresses. | X (page 4) |
| **METHODS** | | |  |
| Eligibility criteria | 5 | Specify the inclusion and exclusion criteria for the review and how studies were grouped for the syntheses. | X (section 2.2) |
| Information sources | 6 | Specify all databases, registers, websites, organisations, reference lists and other sources searched or consulted to identify studies. Specify the date when each source was last searched or consulted. | X (section 2.1) |
| Search strategy | 7 | Present the full search strategies for all databases, registers and websites, including any filters and limits used. | X (appendix A) |
| Selection process | 8 | Specify the methods used to decide whether a study met the inclusion criteria of the review, including how many reviewers screened each record and each report retrieved, whether they worked independently, and if applicable, details of automation tools used in the process. | X (sections 2.2, 2.3) |
| Data collection process | 9 | Specify the methods used to collect data from reports, including how many reviewers collected data from each report, whether they worked independently, any processes for obtaining or confirming data from study investigators, and if applicable, details of automation tools used in the process. | X (section 2.3) |
| Data items | 10a | List and define all outcomes for which data were sought. Specify whether all results that were compatible with each outcome domain in each study were sought (e.g. for all measures, time points, analyses), and if not, the methods used to decide which results to collect. | X (sections 2.3, 2.5, 3.1, 3.2) |
|  | 10b | List and define all other variables for which data were sought (e.g. participant and intervention characteristics, funding sources). Describe any assumptions made about any missing or unclear information. | X (section 3.1) |
| Study risk of bias assessment | 11 | Specify the methods used to assess risk of bias in the included studies, including details of the tool(s) used, how many reviewers assessed each study and whether they worked independently, and if applicable, details of automation tools used in the process. | X (section 2.4, 3.3) |
| Effect measures | 12 | Specify for each outcome the effect measure(s) (e.g. risk ratio, mean difference) used in the synthesis or presentation of results. | X (sections 3.2, 3.4, 3.5) |
| Synthesis methods | 13a | Describe the processes used to decide which studies were eligible for each synthesis (e.g. tabulating the study intervention characteristics and comparing against the planned groups for each synthesis (item #5)). | X (section 2.2) |
|  | 13b | Describe any methods required to prepare the data for presentation or synthesis, such as handling of missing summary statistics, or data conversions. | X (sections 2.3, 2.5) |
|  | 13c | Describe any methods used to tabulate or visually display results of individual studies and syntheses. | X (section 2.5) |
|  | 13d | Describe any methods used to synthesize results and provide a rationale for the choice(s). If meta-analysis was performed, describe the model(s), method(s) to identify the presence and extent of statistical heterogeneity, and software package(s) used. | X (section 2.5) |
|  | 13e | Describe any methods used to explore possible causes of heterogeneity among study results (e.g. subgroup analysis, meta-regression). | X (section 2.5) |
|  | 13f | Describe any sensitivity analyses conducted to assess robustness of the synthesized results. | X (section 2.5) |
| Reporting bias assessment | 14 | Describe any methods used to assess risk of bias due to missing results in a synthesis (arising from reporting biases). | X (section 2.4) |
| Certainty assessment | 15 | Describe any methods used to assess certainty (or confidence) in the body of evidence for an outcome. | NA |
| **RESULTS** | | |  |
| Study selection | 16a | Describe the results of the search and selection process, from the number of records identified in the search to the number of studies included in the review, ideally using a flow diagram. | X (figure 1, section 3.1) |
|  | 16b | Cite studies that might appear to meet the inclusion criteria, but which were excluded, and explain why they were excluded. | X (section 3.1, table S3) |
| Study characteristics | 17 | Cite each included study and present its characteristics. | X (table 1, table 2) |
| Risk of bias in studies | 18 | Present assessments of risk of bias for each included study. | X (table 1, section 3.3) |
| Results of individual studies | 19 | For all outcomes, present, for each study: (a) summary statistics for each group (where appropriate) and (b) an effect estimate and its precision (e.g. confidence/credible interval), ideally using structured tables or plots. | X (table 1, table 2, table S2) |
| Results of syntheses | 20a | For each synthesis, briefly summarise the characteristics and risk of bias among contributing studies. | X (section 3.3) |
|  | 20b | Present results of all statistical syntheses conducted. If meta-analysis was done, present for each the summary estimate and its precision (e.g. confidence/credible interval) and measures of statistical heterogeneity. If comparing groups, describe the direction of the effect. | X (section 3.4, 3.5) |
|  | 20c | Present results of all investigations of possible causes of heterogeneity among study results. | X (section 3.4, 3.5) |
|  | 20d | Present results of all sensitivity analyses conducted to assess the robustness of the synthesized results. | X (section 3.4, 3.5) |
| Reporting biases | 21 | Present assessments of risk of bias due to missing results (arising from reporting biases) for each synthesis assessed. | X (table 1) |
| Certainty of evidence | 22 | Present assessments of certainty (or confidence) in the body of evidence for each outcome assessed. | NA |
| **DISCUSSION** | | |  |
| Discussion | 23a | Provide a general interpretation of the results in the context of other evidence. | X (Section 4) |
|  | 23b | Discuss any limitations of the evidence included in the review. | X (Section 4, page 13) |
|  | 23c | Discuss any limitations of the review processes used. | X (Section 4, page 13) |
|  | 23d | Discuss implications of the results for practice, policy, and future research. | X (section 5) |
| **OTHER INFORMATION** | | |  |
| Registration and protocol | 24a | Provide registration information for the review, including register name and registration number, or state that the review was not registered. | X (section 2) |
|  | 24b | Indicate where the review protocol can be accessed, or state that a protocol was not prepared. | X (section 2) |
|  | 24c | Describe and explain any amendments to information provided at registration or in the protocol. | X |
| Support | 25 | Describe sources of financial or non-financial support for the review, and the role of the funders or sponsors in the review. | X (page 14) |
| Competing interests | 26 | Declare any competing interests of review authors. | X (page 14) |
| Availability of data, code and other materials | 27 | Report which of the following are publicly available and where they can be found: template data collection forms; data extracted from included studies; data used for all analyses; analytic code; any other materials used in the review. | NA |

*From:*  Page MJ, McKenzie JE, Bossuyt PM, Boutron I, Hoffmann TC, Mulrow CD, et al. The PRISMA 2020 statement: an updated guideline for reporting systematic reviews. BMJ 2021;372:n71. doi: 10.1136/bmj.n71. This work is licensed under CC BY 4.0. To view a copy of this license, visit <https://creativecommons.org/licenses/by/4.0/>

**Table S2.** List of excluded works with reason of exclusion.

| **Title** | **First Author** | **Year** | **Decision** | **Reason of exclusion** |
| --- | --- | --- | --- | --- |
| Patterns of Dietary Blood Markers Are Related to Frailty Status in the FRAILOMIC Validation Phase. | Henning et al. | 2023 | excluded | no details on diet |
| Adding Branched-Chain Amino Acids to an Enhanced Standard-of-Care Treatment Improves Muscle Mass of Cirrhotic Patients with Sarcopenia: A Placebo-Controlled Trial. | Hernández-Conde et al. | 2021 | excluded | age, not healthy |
| Untargeted Metabolomic Assay of Prefrail Older Adults after Nutritional Intervention. | Jaroch et al. | 2022 | included |  |
| Identification of pre-frailty sub-phenotypes in elderly using metabolomics. | Pujos-Guillot et al. | 2019 | included |  |
| Plant protein but not animal protein consumption is associated with frailty through plasma metabolites. | Tanaka et al. | 2023 | included |  |
| Metabolomic Profile of Different Dietary Patterns and Their Association with Frailty Index in Community-Dwelling Older Men and Women. | Tanaka et al. | 2022 | included |  |
| Metabolomic profile of different dietary patterns and their association with frailty. | Tanaka et al. | 2022 | excluded | Abstract |
| Dietary patterns, metabolomics and frailty in a large cohort of 120 000 participants. | Yao et al. | 2024 | excluded | Age |
| Plasmatic Hippuric Acid as a Hallmark of Frailty in an Italian Cohort: The Mediation Effect of Fruit-Vegetable Intake. | Brunelli et al. | 2021 | included |  |
| Nutritional Status and Renal Function in Relation to Frailty among the Community-Dwelling Elderly Taiwanese Population. | Chang et al. | 2022 | excluded | no metabolomic methodology |
| Associations of Inflammatory, Metabolic, Malnutrition, and Frailty Indexes with Multimorbidity Incidence and Progression, and Mortality Impact: Singapore Longitudinal Aging Study. | Cheong et al. | 2023 | excluded | no metabolomic methodology, age |
| Intake of vegetables and fruits at midlife and the risk of physical frailty in later life. | Chua et al. | 2024 | excluded | age, not healthy |
| Dietary profiling of physical frailty in older age phenotypes using a machine learning approach: the Salus in Apulia Study. | De Nucci et al. | 2023 | excluded | no metabolomic methodology |
| Urinary phytoestrogen levels and frailty in older american women of the national health and nutrition examination survey (NHANES) 1999-2002: A cross-sectional study. | Eichholzer et al. | 2013 | excluded | no info about diet, age |
| Mediterranean diet intervention alters the gut microbiome in older people reducing frailty and improving health status: The NU-AGE 1-year dietary intervention across five European countries. | Ghosh et al. | 2020 | excluded | no metabolomic methodology |
| Association between Dietary Flavonoid Intake and the Likelihood of Frailty in Middle-Aged and Older Adults: A Population-Based Analysis from the National Health and Nutrition Examination Survey (NHANES). | Guo et al. | 2024 | excluded | frail population |
| Association of a mixture of phthalates and phenols with frailty among middle-aged and older adults: A population-based cross-sectional study. | Guo et al. | 2023 | excluded | age |
| Frailty severity and cognitive impairment associated with dietary diversity in older adults in taiwan. | Huang et al. | 2021 | excluded | no metabolomic methodology |
| Frailty, nutrition-related parameters, and mortality across the adult age spectrum. | Jayanama et al. | 2018 | excluded | no metabolomic methodology |
| Dietary Fat Composition and Frailty in Oldest-Old Men. | Jyväkorpi et al. | 2020 | excluded | not research article |
| Circulating cell-free DNA in health and disease — the relationship to health behaviours, ageing phenotypes and metabolomics. | Kananen et al. | 2023 | excluded | age |
| Frailty is characterized by biomarker patterns reflecting inflammation or muscle catabolism in multi-morbid patients. | Kochlik et al. | 2023 | excluded | not healthy |
| Associations of fat-soluble micronutrients and redox biomarkers with frailty status in the FRAILOMIC initiative. | Kochlik et al. | 2019 | excluded | not adjusted for dietary intake or specific dietary pattern |
| Association between co-exposure to phenols, phthalates, and polycyclic aromatic hydrocarbons with the risk of frailty. | Li et al. | 2023 | excluded | no diet info and wrong age |
| Dietary inflammatory index, dietary total antioxidant capacity, and frailty among older Chinese adults. | Li et al. | 2024 | excluded | no biomarkers measured with metabolomic analysis |
| Metabolites associated with vigor to frailty among community-dwelling older black men. | Marron et al. | 2019 | excluded | no diet details, pop non healthy |
| A metabolite composite score attenuated a substantial portion of the higher mortality risk associated with frailty among community-dwelling older adults. | Marron et al. | 2021 | excluded | no details on diet |
| Oxylipins Associated with D3-Creatine Muscle Mass/Weight and Physical Performance among Community-Dwelling Older Men. | Marron et al. | 2022 | excluded | no details on diet |
| Metabolomic characterization of vigor to frailty among community-dwelling older Black and White men and women. | Marron et al. | 2024 | excluded | no details on diet |
| Vitamin and carotenoid status in older women: Associations with the frailty syndrome. | Michelon et al. | 2006 | excluded | no metabolomic methodology |
| Adherence to the Mediterranean-style diet and high intake of total carotenoids reduces the odds of frailty over 11 years in older adults: Results from the Framingham Offspring Study. | Millar et al. | 2022 | excluded | no metabolomic methodology |
| Functional frailty, dietary intake, and risk of malnutrition. Are nutrients involved in muscle synthesis the key for frailty prevention? | Moradell et al. | 2021 | excluded | no metabolomic methodology |
| Intake of flavonoids and odds of frailty onset in adults in the framingham offspring cohort. | Nguyen et al. | 2021 | excluded | Abstract |
| Effects of therapeutic lifestyle change diets high and low in dietary fish-derived FAs on lipoprotein metabolism in middle-aged and elderly subjects. | Ooi et al. | 2012 | excluded | age |
| Metabolites as frailty biomarkers in older adults. | Pan et al. | 2021 | excluded | comment |
| Lack of Association between Insufficient Intake of Multiple Vitamins and Frailty in Older Adults Who Consume Sufficient Energy and Protein: A Nationwide Cross-Sectional Study. | Park et al. | 2024 | excluded | no metabolomic methodology |
| Patterns of circulating fat-soluble vitamins and carotenoids and risk of frailty in four European cohorts of older adults. | Pilleron et al. | 2019 | excluded | no details on diet |
| Association of habitual dietary resveratrol exposure with the development of frailty in older age: the Invecchiare in Chianti study. | Rabassa et al. | 2015 | excluded | no details on diet |
| Guava Fruit and Acacia pennata Vegetable Intake Association with Frailty of Older Adults in Northern Thailand. | Ruangsuriya et al. | 2022 | excluded | no metabolomic methodology |
| Lower nutritional status and higher food insufficiency in frail older US adults. | Smit et al. | 2013 | excluded | no metabolomic methodology |
| The relationship between urinary total polyphenols and the frailty phenotype in a community-dwelling older population: The InCHIANTI Study. | Urpi-Sarda et al. | 2015 | excluded | no metabolomic methodology |
| Dietary education with customised dishware and food supplements can reduce frailty and improve mental well-being in elderly people: A single-blind randomized controlled study. | Wu et al. | 2018 | excluded | not healthy |
| The Association Between the Composite Dietary Antioxidant Index and Frailty Symptoms: Mediating Effects of Oxidative Stress. | Wu et al. | 2024 | excluded | no metabolomic methodology |

**Table S3.** List of statistical tests used in the included studies.

| **Reference** | **Statistical test** | **p-value** |
| --- | --- | --- |
| Jaroch 2022 | Mann–Whitney Pearson’s chi-squared test one-way ANOVA | p≤0,05 |
| Pujos-Guillot 2019 | Wilcoxon test or Fisher’s exact tests Two-ways ANOVA multivariate logistic regression | p≤0,05 |
| Tanaka 2023 | One-way ANOVA chi-square tests multiple linear regression model mediation analysis | p≤0,05 |
| Tanaka 2022 | Linear regression model mediation analysis | p≤0,05 |
| Brunelli 2021 | Kruskal–Wallis test Mann–Whitney U test Baron and Kenny method for mediation analysis binary logistic regression | p≤0,05 |
